# Complete Heart Block: A Rare Initial Presentation of Epstein-Barr Virus-Induced Myocarditis

**DOI:** 10.7759/cureus.77884

**Published:** 2025-01-23

**Authors:** Ridha Umar, Kinza Moin, Tarab Iqbal, Resshme K Sudha, Thiagarajan Jaiganesh

**Affiliations:** 1 Medical School, University of Sharjah, Sharjah, ARE; 2 Department of Emergency Medicine, Tawam Hospital, Al Ain, ARE

**Keywords:** complete heart block, ebv, epstein bar virus, heart failure, myocarditis, viral illness

## Abstract

Myocarditis, a rare but serious condition often caused by viral infections, rarely manifests with cardiac involvement in healthy individuals infected with Epstein-Barr virus (EBV). We present a case of a 51-year-old diabetic presenting with exertional dyspnoea, pleuritic chest pain, and intermittent fever. Investigations revealed elevated C-reactive protein (CRP), brain natriuretic peptide (BNP), high sensitive troponin, creatine kinase (CK) levels, and complete heart block on ECG. Echocardiography showed reduced ejection fraction and cardiac MRI confirmed myocarditis. Epstein-Barr virus was confirmed as the underlying cause. He was treated with prednisone, acyclovir, and a permanent pacemaker. Due to serious complications associated with myocarditis such as acute heart failure and arrhythmias, it is imperative to emphasize the importance of early recognition and timely intervention of atypical presentations.

## Introduction

Myocarditis is an inflammatory condition of the myocardium caused by both infectious and non-infectious factors. Often considered a diagnosis of exclusion, its presentation can mimic other cardiac pathologies [1]. In the Western world, viral infections are the most common cause, with Coxsackievirus and Parvovirus being the primary culprits. However, myocarditis can also arise from non-viral aetiologies, including autoimmune disorders such as systemic lupus erythematosus and sarcoidosis, bacterial infections like Staphylococcus aureus or Streptococcus species, parasitic infections such as Trypanosoma and Toxoplasma, and exposure to toxins. The prevalence of myocarditis caused by Epstein-Barr virus (EBV) is reported to be approximately 2% or less [2]. We present the case of a 51-year-old male diagnosed with myocarditis secondary to Epstein-Barr virus, manifesting as complete heart block and symptoms of acute heart failure that necessitated the implantation of a permanent pacemaker.

## Case presentation

A 51-year-old man with a 10-year history of diabetes was referred from a private clinic to the emergency department of a tertiary care hospital. He presented with a five-day history of progressive exertional shortness of breath, accompanied by mild pleuritic chest pain and intermittent fever over the past week. He denied any syncopal episodes, cough, sore throat, weight loss, excessive sweating, recent travel, or contact with sick individuals. 

On physical examination, the patient appeared comfortable at rest. Vital signs showed bradycardia with a heart rate of 50 beats per minute, blood pressure of 144/69 mmHg, respiratory rate of 14 breaths per minute, and oxygen saturation of 98% on room air. On chest auscultation, bibasilar crackles were present, however, there were no signs of jugular venous distension, or peripheral oedema. Heart sounds were normal on auscultation. No rash, tonsillar exudates, oropharyngeal erythema, lymphadenopathy, hepatosplenomegaly, radio-radial delay or calf tenderness was present.

**Figure 1:**
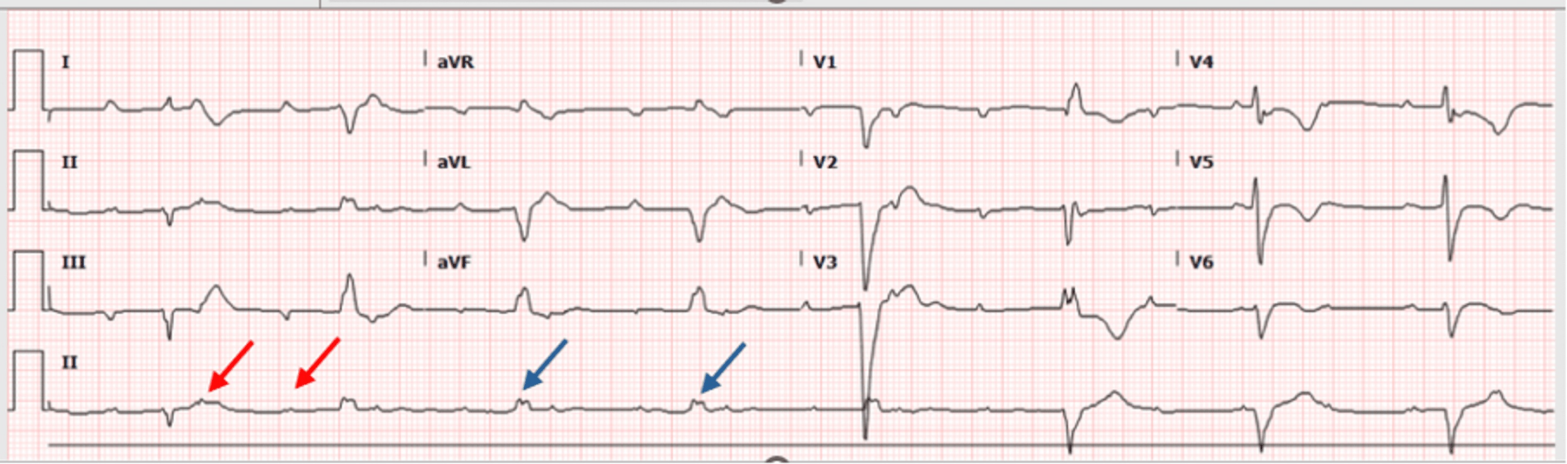
Atrioventricular (AV) dissociation with regular P-P (red arrows) and R-R intervals (blue arrows).

**Figure 2:**
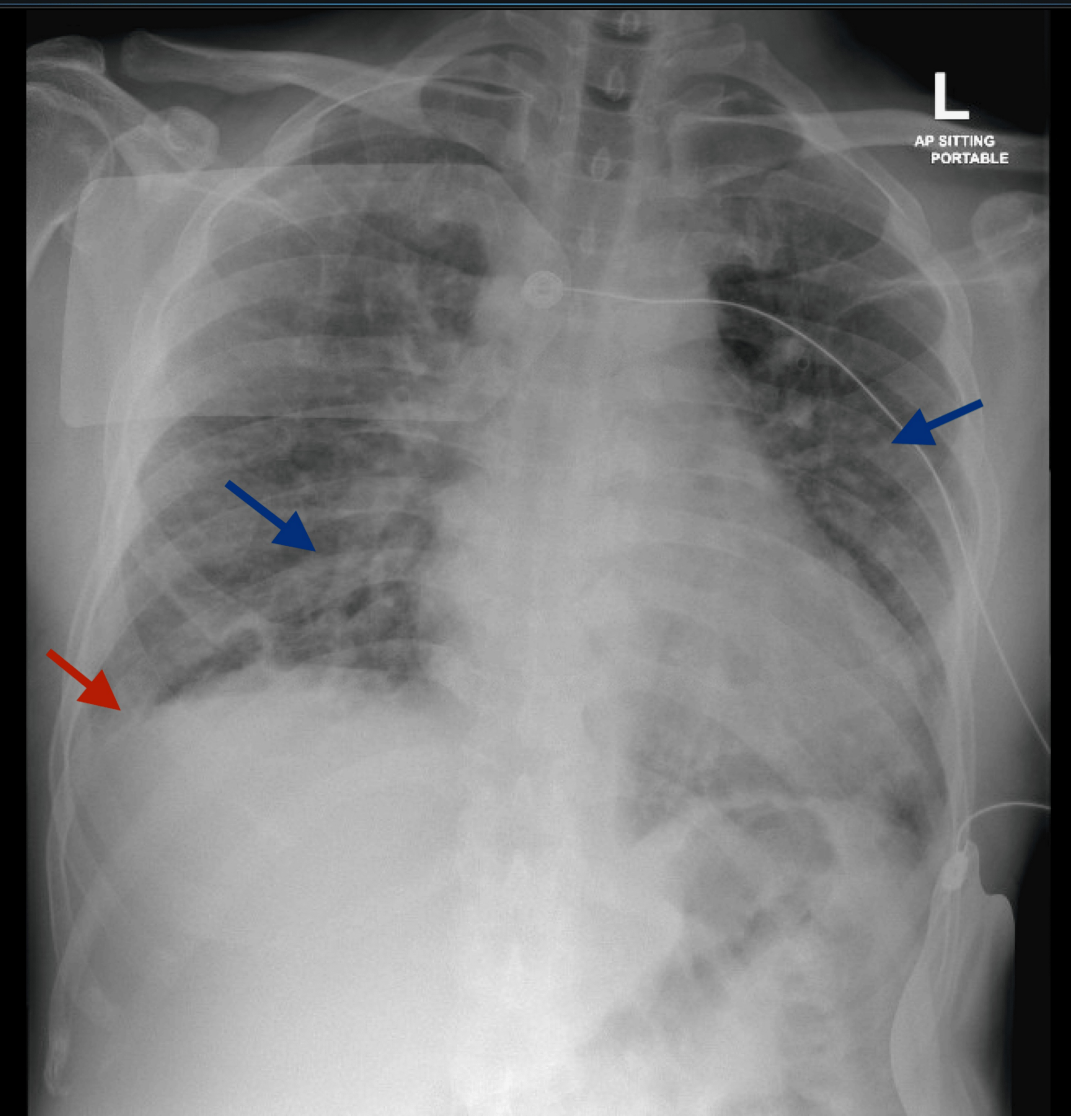
CXR showing increased broncho-vascular markings (blue arrows) and right sided pleural effusion (red arrow) CXR - Chest X-Ray

Initial laboratory results revealed no leukocytosis or electrolyte imbalances but showed an elevated C-reactive protein (CRP) level of 40 mg/L (<5 mg/L) and elevated creatinine kinase of 250 U/L (<170 U/L). The brain natriuretic peptide (BNP) was significantly elevated at 2889 pg/mL (<100 pg/ml), and high sensitivity cardiac troponin level was 50 ng/L (<14 ng/L), indicating myocardial injury. A random blood glucose level revealed 15 mmol/L (<11 mmol/L), with normal pH, bicarbonate and anion gap. Liver function and renal function tests were unremarkable. In the emergency department, an electrocardiogram (ECG) demonstrated a complete heart block (Figure 1), and a chest X-ray revealed bilateral prominent broncho-pulmonary markings and right-sided pleural effusion (Figure 2). The echocardiogram showed a reduced ejection fraction of 45%. Both the right and left ventricles were normal in size, with no evidence of clots or pericardial effusion.

The patient received symptomatic treatment in the emergency department and was subsequently admitted to the Coronary Care Unit (CCU) for serial troponin measurements, ECG monitoring, and elective angiography. Left heart catheterization and coronary angiography revealed no blockage or stenosis in the left main coronary artery, left anterior descending artery, left circumflex artery, and right coronary artery.

**Figure 3:**
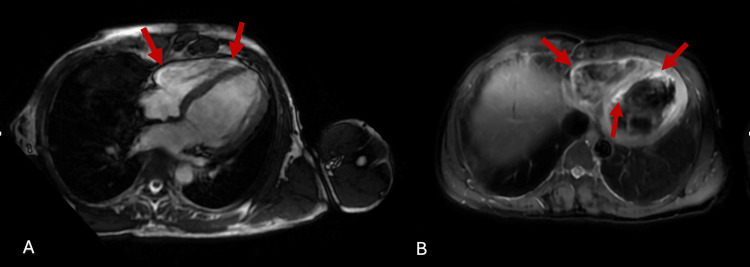
Cardiac MRI - Red arrows indicate right ventricular wall, apical (A and B), and septal (B) segments fibrosis and myocardial edema. MRI - Magnetic resonance imaging

Diagnostic workup to determine the underlying cause of high-degree atrioventricular (AV) block was performed which included Brucella, hepatitis, cytomegalovirus, influenza, parainfluenza, adenovirus, Mycoplasma, metapneumovirus, COVID-19, syphilis, Toxoplasma, Legionella, Borrelia, sarcoidosis, Lyme disease, autoimmune markers including anti-nuclear antibodies (ANA), anti-double stranded deoxyribonucleic acid (dsDNA), extractable nuclear antigen (ENA), anti-neutrophil cytoplasmic antibodies (cANCA), and perinuclear anti-neutrophil cytoplasmic antibodies (pANCA), were all found to be negative. However, IgM antibodies to Epstein-Barr virus nuclear and capsid antigens were detected along with a positive heterophile monospot test indicating an active EBV infection in our patient. Cardiac MRI findings revealed active myocarditis with myocardial fibrosis mainly involving the right ventricular wall, septal and apical region along with myocardial edema (Figure 3).

**Figure 4:**
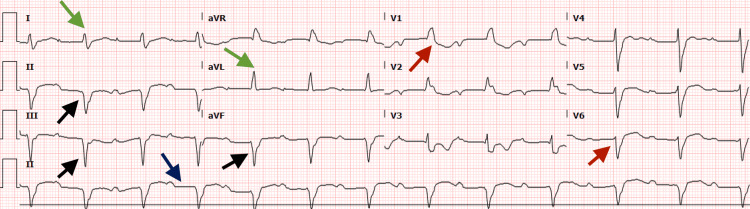
ECG shows trifascicular block including atrioventricular block (blue arrow), right bundle branch block (red arrows), and left anterior fascicular block (green and black arrows) ECG - Electrocardiogram

Based on the clinical presentation, serology results, and cardiac MRI findings, the patient was initiated on prednisone at a dose of 50 mg. Given that the subsequent ECG showed a trifascicular block (Figure 4), an urgent decision was made to implant a permanent pacemaker. 

**Figure 5:**
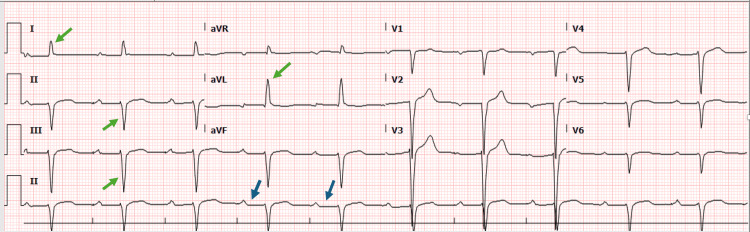
ECG at the time of discharge showing first degree AV block (blue arrow) and left anterior fascicular block (green arrows) ECG - Electrocardiogram AV - Atrioventricular

Our patient had a nine-day hospitalization during which he was treated with furosemide 40 mg for his heart failure symptoms. Myocarditis was initially managed with acyclovir, predisolone 50 mg which was later tapered to 10 mg. He was also started on insulin glargine and aspart for glycemic control. The patient's symptoms resolved by day 9, and he was discharged with a stable heart rate of 65 beats per minute with ECG showing first-degree heart block along with left anterior fascicular block (Figure 5), and static serial troponin levels. He was followed up in the cardiology clinic after one week where he reported no symptoms and his pacemaker functioned as expected. 

## Discussion

Epstein-Barr virus, also known as human herpes virus-4 (HHV-4), belongs to the Herpesviridae family. It is a double-stranded DNA virus that primarily infects B-lymphocytes and is notably the first virus identified to induce tumour formation in humans, including Burkitt’s lymphoma, nasopharyngeal carcinoma, and gastric carcinoma [3]. The clinical presentation of EBV infection varies widely; some patients remain asymptomatic, while others exhibit nonspecific symptoms such as fever, fatigue, malaise, rash, or petechiae. In more severe cases, systemic manifestations may occur, including sore throat, tonsillar enlargement, lymphadenopathy, hepatomegaly, and splenomegaly [4].

Although cardiovascular involvement is rare, it represents a serious complication of EBV infection. Cardiac manifestations of EBV include myocarditis, coronary artery aneurysms, valvular heart diseases, coronary artery dilation, and complete heart block [2]. While the exact pathophysiological mechanisms remain unclear, several theories have been proposed. EBV may induce host cell lysis, disrupting cellular function, and trigger the production of inflammatory cytokines, alongside the recruitment of immune cells, contributing to tissue damage and inflammation [5].

Patients with myocarditis present with a wide range of symptoms, including chest pain, dyspnoea, tachypnoea, palpitations, fatigue, fever, and tachycardia. Physical examination findings may include rales, an S3 gallop, and peripheral oedema, often mimicking congestive heart failure. Myocarditis caused by the Epstein-Barr virus may result in elevated cardiac biomarkers, C-reactive protein, and creatine kinase levels; however, these laboratory findings are typically non-specific. The prognosis tends to be worse in those presenting with chest pain or symptoms resembling heart failure [6]. The combination of elevated troponin, creatine kinase, and C-reactive protein levels, along with a history of exertional dyspnea and physical examination findings of bibasilar crackles, strongly suggested acute heart failure secondary to myocarditis in our patient.

The diagnosis of EBV can be established using serological tests that detect the presence of EBV-specific antibodies in the serum. These tests help determine and classify the infection status of the patient. Several genes within the EBV genome encode antigens utilized in serological assays, including viral capsid antigens (VCAs), early antigens (EAs), and Epstein-Barr nuclear antigens (EBNAs). Additionally, the Paul-Bunnell monospot test, also known as the heterophile antibody test, is commonly used for the diagnosis of EBV infection. Our patient had a positive EBV IgM assay and heterophile monospot test [7].

ECG findings in myocarditis often include non-specific ST segment elevations or depressions, along with T wave inversions. In rare cases, an AV block may be observed. Echocardiography and angiography are valuable for ruling out other potential cardiac complications associated with EBV infection. Cardiac magnetic resonance imaging (CMRI), a non-invasive diagnostic tool, has gained prominence for detecting acute myocarditis due to its ability to visualize myocardial inflammation and necrosis [8]. CMRI demonstrates a sensitivity of 80-90%, making it highly effective in diagnosing myocarditis, while its specificity of 70-85% allows for moderate reliability in distinguishing myocarditis from other cardiac conditions [9]. However, endomyocardial biopsy remains the gold standard [10]. A high-degree AV block on ECG and evidence of myocardial inflammation and fibrosis on CMRI strongly indicated acute myocarditis in our patient. An endomyocardial biopsy was not performed due to the patient’s refusal.

The treatment of EBV myocarditis involves three primary components: antiviral therapy, anti-inflammatory medications, and management of complications associated with cardiovascular involvement. During the acute phase, viral replication of EBV can be controlled with antiviral agents such as acyclovir or ganciclovir. To reduce inflammation, corticosteroids, and immunosuppressive medications, including azathioprine, mycophenolate mofetil, and methotrexate, may be utilized. Management of myocarditis focuses on monitoring cardiac rhythm and employing heart failure treatment strategies. Low cardiac output is managed by inotropic agents, such as dobutamine or dopamine and vasopressors like epinephrine or norepinephrine to increase the cardiac output and vascular tone, respectively. For significant arrhythmias or conduction abnormalities, pacemakers may be required to ensure proper cardiac rhythm and optimal heart function [8]. During his stay, he was treated with acyclovir, prednisolone, and furosemide along with strict control of diabetes with insulin. Given his high-degree AV block, a pacemaker was inserted. 

The patient was deemed fit for discharge following a period of observation and was prescribed a pharmacological regimen, including tapering steroids, angiotensin-converting enzyme inhibitors, and diuretics. The final diagnosis was acute Epstein-Barr virus-related myopericarditis.

## Conclusions

In conclusion, although cardiac involvement in Epstein-Barr virus infections is rare, it should be considered in cases of unexplained acute heart failure or arrhythmias. Early recognition, supported by diagnostic tools such as cardiac MRI and biopsy, is essential for confirming myocarditis and guiding appropriate treatment. Complications such as complete heart block and heart failure underscore the necessity for tailored therapeutic approaches. This case highlights the importance of comprehensive etiological evaluation and timely intervention to improve outcomes in myocarditis with uncommon presentations. 
